# Use of slow-release plant infochemicals to control aphids: a first investigation in a Belgian wheat field

**DOI:** 10.1038/srep31552

**Published:** 2016-08-17

**Authors:** Haibo Zhou, Longsheng Chen, Yong Liu, Julian Chen, Frédéric Francis

**Affiliations:** 1 State Key Laboratory for Biology of Plant Disease and Insect Pests, Institute of Plant Protection, Chinese Academy of Agricultural Sciences, Beijing, 100193, PR China; 2 Functional and Evolutionary Entomology, Gembloux Agro-Bio Tech, University of Liege, Gembloux, 5030, Belgium; 3 Anhui Academy of Science and Technology, Heifei, 230031, PR China; 4 Anhui Academy of Applied Technology, Heifei, 230088, PR China; 5 College of Plant Protection, Shandong Agricultural University, Taian, 271018, China

## Abstract

Using infochemicals to develop a push–pull strategy in pest control is a potential way to promote sustainable crop production. Infochemicals from plant essential oils were mixed with paraffin oil for slow release in field experiments on wheat to control the population density of cereal aphids and to enhance their natural enemies. (*Z*)-3-Hexenol (Z3H) attracted *Metopolophum dirhodum* and *Sitobion avenae*, the predominant species on wheat in Belgium, and may be a useful infochemical for aphid control by attracting aphids away from field plots. Release of (*E*)-β-farnesene (EBF) or a garlic extract (GE) led to a significant decrease in the abundance of wheat aphids. The main natural enemies of cereal aphids found were lacewings (47.8%), hoverflies (39.4%), and ladybirds (12.8%). Ladybird abundance varied little before the end of the wheat-growing season. Our results suggest that these chemicals can form the basis of a “push–pull” strategy for aphid biological control, with GE and EBF acting as a pest- and beneficial-pulling stimulus and Z3H for aphid pulling.

Among aphid species, the grain aphid [*Sitobion avenae* (Fabricius)], bird cherry-oat aphid [*Rhopalosiphum padi* (L.)], and rose-grain aphid [*Metopolophium dirhodum* (Walker)] are considered the major pests that infest cereal crops as a result of feeding on phloem and transmitting viruses^1^,^2^, particularly on winter wheat (*Triticum aestivum* L. [Poaceae]) in Europe^3^. Aphid populations often fluctuate greatly from year to year^4^ and are affected by a range of biotic and abiotic factors^5^.

Because of the urgent need for sustainable agricultural methods and reduced reliance on pesticide use, more integrated pest management studies are focusing on the ecological effect of volatiles released by plants on herbivores and their natural enemies^6^,^7^,^8^,^9^,^10^,^11^,^12^,^13^,^14^. Several studies on volatiles under natural conditions have demonstrated their applicability for enhancing natural enemy abundance on strawberry (*Fragaria* ×*ananassa*)^7^, cotton (*Gossypium* spp.)^12^, hops (*Humulus lupulus*)^8^ and grapes (*Vitis vinifera*)^9^ and for reducing pest populations in wheat (*Triticum aestivum*)^6^,^15^ and barley (*Hordeum vulgare*)^16^.

While attracting natural enemies of these herbivores^17^, volatiles emanating from herbivore-infested plants may also stimulate plant defense against herbivores and serve as recognition cues between two or more individuals^18^. Dicke *et al*. (1987, 1990) presented the first convincing evidence that the active release of volatiles by herbivore-infested plants attracts natural enemies of the attackers^19^,^20^. Aphid behaviour is also affected by a density mechanism that is mediated by volatile compounds released at the feeding site when their density exceeds a certain threshold^16^. A further study revealed that these volatiles could increase the sensitivity of aphids to disturbance and promote mobility of nonsettled individuals^21^.

Because they are a natural emission from plants, essential oils do not pose the toxicity problems of pesticides to animals and the environment^15^,^22^. Plant semiochemicals should be considered as potential reliable infochemicals in relation to to repelling pests and attracting natural enemies of these pests. Their long-distance effects and easy production and manipulation make these molecules very good prospects for use with crops by spraying or mixing with a slow-releasing carrier to repel insect feeding or ovipositing from host plants and/or to guide them to nonhosts^23^.

Japanese termite (*Reticulitermes speratus*)^24^, sciarid fly [*Lycoriella ingénue* (Dufour)]^22^ and pine wood nematode (*Bursaphelenchus xylophilus*)^25^ were repelled by a garlic (*Allium sativum*) extract (GE), providing direct evidence that strongly aromatic crops such as garlic, can act as an olfactory camouflage by masking normal host-locating or feeding cues of insects (Perrin and Phillips, 1978). (*E*)-β-Farnesene (EBF), an important volatile sesquiterpene that occurs widely in both plant and animal taxa, such as aphids^26^ and peppermint (*Mentha* ×*piperita* L.)^27^, is an effective kairomone for ladybirds^28^,^29^,^30^, lacewings^31^ and hoverflies^32^. It is proven to be the main or only component of the aphid alarm pheromones for many pest aphids^33^,^34^,^35^,^36^,^37^.

Herbivore-induced volatiles (HIVs), for example, (*Z*)-3-hexenol (Z3H), can directly affect the physiology and behavior of herbivores^38^. Z3H has been demonstrated to attract *Agrilus planipennis* in the laboratory and field^39^,^40^ and the fruit moth *Cydia molesta*^41^. Although it has been difficult to determine whether Z3H is an attractant or a repellent, accumulating evidence suggests that Z3H is an important plant-derived infochemical that can modulate the behavior of herbivorous insects and that the release of Z3H induces defensive responses in the plants against insect pests^38^.

Extensive evidence implies that nearly all herbivorous insects and their natural enemies can perceive and positively respond to plant volatiles. In this investigation, the essential oils of plant volatiles (EBF, GE and Z3H) were released in a wheat field to assess their potential for managing aphid populations by reducing aphid abundance and promoting their natural enemies.

## Materials and Methods

### Experimental design of field studies

**Figure 1:**
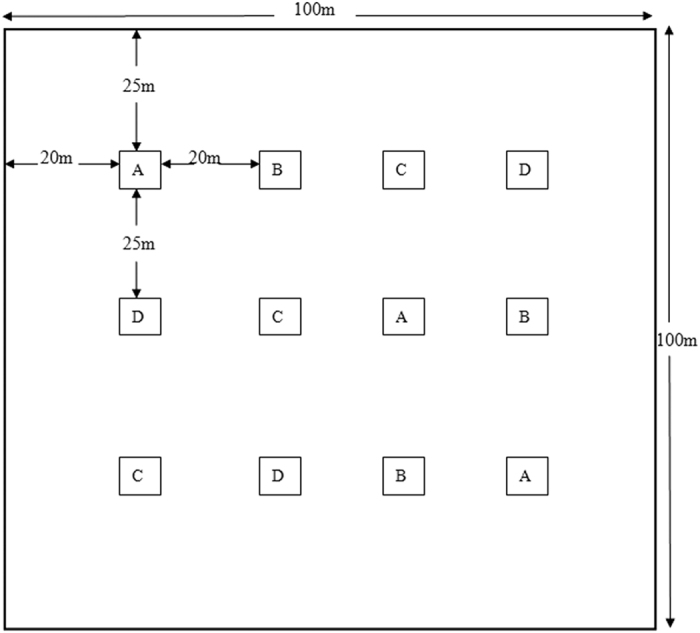
Layout of traps within experimental field. (**A**) Paraffin oil, (**B**) (*E*)-β-farnesene, (**C**) garlic extract, (**D**) (*Z*)-3-hexenol.

In the experimental fields of Gembloux Agro-Bio Tech, University de Liege, Namur Province of Belgium (50 °33″ N, 4 °42″ E) in 2011, traps were set out as shown in Fig. 1. The trial consisted of four treatments in the wheat field: (1) only paraffin oil (PO) as the control, (2) (*E*)-β-farnesene release (EBF), (3) garlic extract release (GE), (4) (*Z*)-3-hexenol release (Z3H). Those extracts were provided by Prof. Frédéric Francis (Gembloux Agro-Bio-Tech., Universite de Liege). Single yellow trap sticks with the releasers were placed 20 m apart in a latin square design with 3 replicates per treatment (12 releasers and 12 traps total). Wheat (cv. Tybalt) was planted in 20-cm-apart rows at 350 seeds/m^2^ on 18 February 2011. No insecticides or herbicides were used in the whole experimental area.

### Assessment of insect abundance and diversity

Yellow traps (26 cm diameter 10 cm depth) that are frequently used to monitor insects in fields^42^ were attached to crabsticks and placed 10 cm above the surface of the wheat plants. Each trap was filled with water and a few drops detergent. Every 7 days, 100 μL of (*E*)-β-farnesene, garlic extract or (*Z*)-3-hexenol solution formulated in paraffin oil (for slow release of the infochemcial) were deposited on a 1-cm-diameter rubber septum that was placed on the top of the trap stick; 76 μg of EBF is released from the formulation over 7 days at 20 °C, 65% relative humidity and air flow of 0.5 litre/min (Dr. S. Heuskin, unpublished data). A similar release rate was applied to the other tested semiochemicals. The slow releasers were first placed in the wheat field at the jointing stage on 4 May.

Traps were emptied and reset at 7-day intervals between 11 May to 29 June. Trap contents were decanted through a 1-mm-mesh sieve and transferred to 70% ethanol in 50-mL plastic vials. In the laboratory, aphids and their natural enemies were sorted and identified to species, and the abundance of each species was recorded.

Aphid abundance in the traps was compared every 7 days to the aphid density determined by visual observation on 20 randomly selected wheat tillers.

### Statistical analyses

For all parametric tests, a data sqrt (n + 1) transformation was applied to stabilize the variance. Population densities of insects were compared among the infochemical releaser tests using a one-way analysis of variance (ANOVA)^43^, followed by Tukey’s honestly significant difference (HSD) test.

## Results

### Abundance and diversity of aphids after exposure to infochemicals

**Figure 2:**
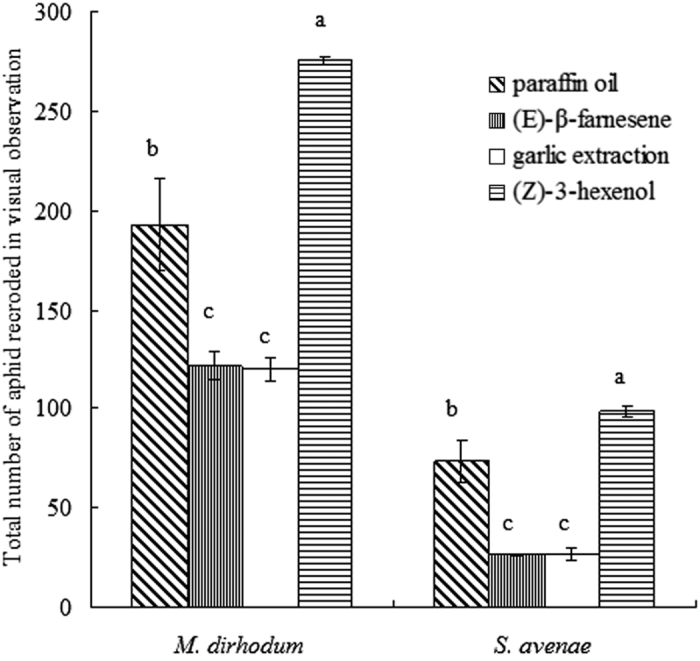
Total number of aphids (mean ± SEM) found in field observations according to infochemical released. Different letters indicate a statistically significant difference between the individual treatments at *P* < 0.05.

*M. dirhodum* and *S. avenae* were the predominant species on wheat, and Z3H was the most attractive to these aphids. EBF and GE repelled aphids significantly within wheat fields. Trapping numbers and visual counts of aphid were consistent. *M. dirhodum* was far more abundant than *S. avenae* in observations and traps (Table 1 and Fig. 2). In addition, several wheat nontarget aphid species were recorded in traps: *Cavariella aegopodii* (Scopoli)*, Aphis fabae* (Scopoli)*, Macrosiphum euphorbiae* (Thomas)*, Myzus persicae* (Sultzer)*, Rhopalosiphum maidis* (Fitch)*, Cavariella ihedbaldi, Nasonovia ribisnigri* (Mosley)*, Phyllaphis fagi* (Linnaeus)*, Chaitophorus* spp. and *Capitophorus* spp.

**Figure 3:**
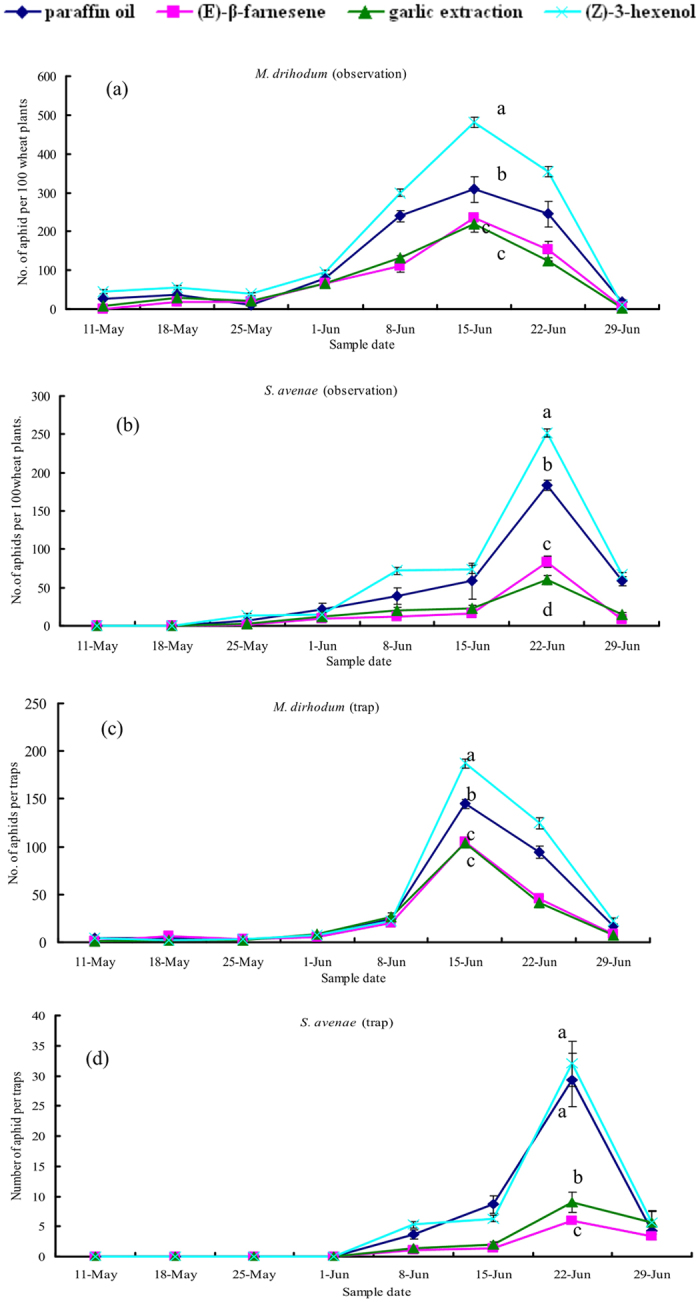
Number of aphids (mean ± SEM) trapped or counted in observations in wheat fields during different treatments in 2011. Different letters indicate a statistically significant difference between individual treatments at *P* < 0.05.

According to visual observations and trapping, the population dynamics of *M. dirhodum* and *S. avenae* in each treatment followed the same trend on growing wheat, with increasing population densities that peaked on 15 June and 22 June, respectively (Fig. 3). Based on visual observations in the field, Z3H attracted mainly *M. dirhodum* for both the highest peak value and total during the whole observation period, whereas EBF and GE repelled aphids (Peak: *F*_3,8_ = 18.95, *P* < 0.01; Total: *F*_3,8_ = 34.45, *P* < 0.01). Similarly, significantly fewer *S. avenae* were found with EBF and GE releasers compared with the control PO (Peak: *F*_3,8_ = 89.30, *P* < 0.01; Total: *F*_3,8_ = 45.55, *P* < 0.01).

Consistent with the results of visual observations, both peak and total abundance of *M. dirhodum* in traps was higher with Z3H and lower with EBF and GE releasers during the experiment (Peak: *F*_3,8_ = 56.30, *P* < 0.01; Total: *F*_3,8_ = 86.27, *P* < 0.01). The highest abundance of *S. avenae* was found in traps with Z3H. EBF and GE releasers were also found to repel *S. avenae* as evidenced by both the peak period and the total during the experiment (Peak: *F*_3,8_ = 56.30, *P* < 0.01; Total: *F*_3,8_ = 86.27, *P* < 0.01). The consistency of the data obtained from visual observations and trapping confirmed the infochemical results for *M. dirhodum* and *S. avenae*.

### Abundance and diversity of natural aphid enemies in response to the infochemical released

The main natural enemies of cereal aphids found in the trials in order of abundance were lacewings (47.8%), hoverflies (39.4%) and ladybirds (12.8%). Of the predatory species, *E. balteatus*, *C. carnea* and *H. axyridis* were the predominant species on wheat. On the basis of total number of aphidophagous species attracted, EBF, GE and Z3H attracted more than the control PO did (Table 1). Not all the collected hoverflies were aphidophagous species (*Eristalis pertinax, Helophilus trivitatus, Cheilosia* spp.*, Eristalis tenax, Eristalis arbustorum*). The aphid predators and their diversity are presented in Table 1.

**Figure 4:**
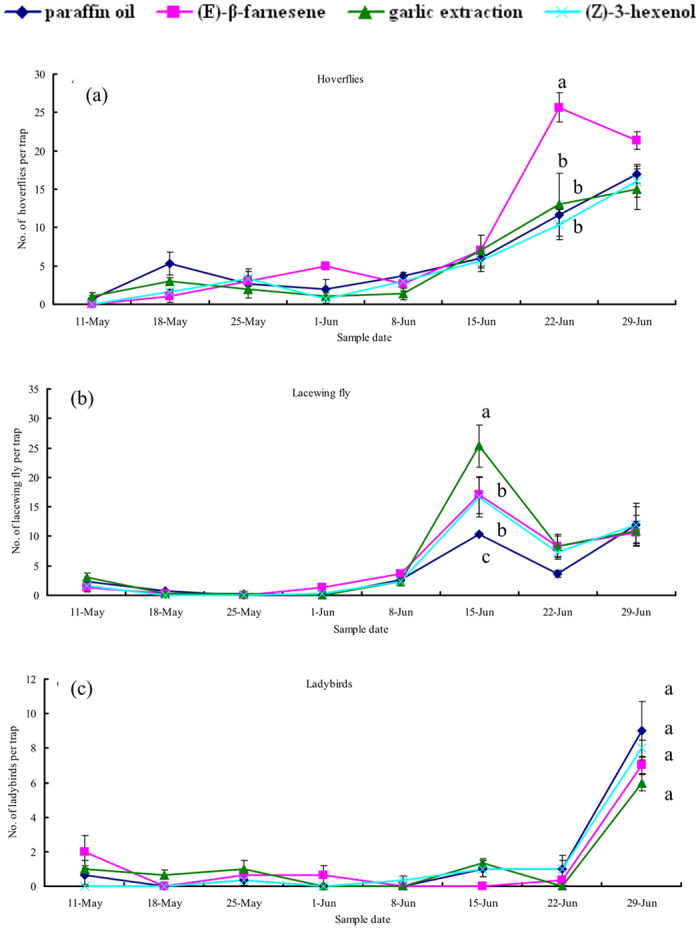
Number of natural enemies (mean ± SEM) trapped during the different treatments in wheat fields in 2011. Different letters indicate a statistically significant difference between individual treatments at *P* < 0.05.

The hoverfly population density had reached its peak by 29 June (Fig. 4(a)). Before this peak, hoverfly density did not differ among the tested infochemicals. After the peak, hoverfly density in response to EBF releases was significantly higher than with Z3H (*F*_3,8_ = 4.46, *P* < 0.05). No significant difference in total hoverfly abundance among treatments was detected (*F*_3,8_ = 1.64, *P* = 0.26).

The number of lacewings peaked in all treatments on 15 June coincident with the peak of *M. dirhodum* (Fig. 4(b)). The population density of lacewings in each treatment was low before 8 June. No significant difference in total lacewing abundance among treatments was detected (*F*_3,8_ = 1.25, *P* = 0.36).

Finally, ladybird population dynamics did not vary significantly among treatments before 22 June (Fig. 4(c)). Moreover, the ladybird population peaked in all treatments at the end of the wheat season when the aphid population declined rapidly in the field. No significant difference in the abundance of ladybirds among treatments was detected either at the peak period or for the total numbers during the experiment (Peak: *F*_3,8_ = 1.92, *P* = 0.21; Total: *F*_3,8_ = 0.52, *P* = 0.68).

## Discussion

The densities of cereal aphids and their natural enemies in wheat were significantly influenced by the test infochemical releasers, mainly with EBF and GE, supporting the view that these volatiles play a significant role in the behavioural ecology of aphids and demonstrating the potential use of the volatiles in pest control. As reviewed by Kunert *et al*.^44^, several factors could contribute to the low abundance of *M. dirhodum* and *S. avenae* in the EBF-release plots. First, EBF emission may directly prevent aphid settling because wild potato (*Solanum berthaultii*) repels the green peach aphid (*Myzus persicae*) by emitting EBF^45^. EBF might also reduce aphid growth rate by disrupting feeding^46^ or by inducing wing formation and reducing aphid population size^47^,^48^. Since winged offspring leave their host plant before starting reproduction, plants that produce EBF could reduce aphid colonization^49^,^50^. Under natural conditions, plants emit infochemical as signals in response to attack by insect herbivores that recruit natural enemies of the herbivores^51^; thus, EBF release in plots might primarily improve the efficiency of the natural enemies in locating their prey. This hypothesis is supported by the results of our study that population densities of hoverflies were higher when EBF release was at its peak. Nevertheless, there were some exceptions to the influence of EBF on lacewings and ladybirds in our investigation. The amount of infochemical in releasers may determine the probability of predator response. Shiojiri *et al*.^52^ showed that seedlings of a cabbage variety attracted more parasitoids (*Cotesia glomerata*) when there were more herbivores on the plant. Further study is needed to demonstrate and clarify the mechanism for this phenomenon.

Aphids perceive the host plant and avoid nonhosts by sensing volatile cues^53^. Garlic plants are not hosts to cereal aphids, so a garlic extract is likely to be unsuitable for aphids. Indeed, population densities of *M. dirhodum* and *S. avenae* were significantly lower in GE-release plots than in the PO control plots. Also worth mentioning is that GE significantly attracted more lacewings than did the PO plots. Moreover, GE did not negatively influence field populations of hoverflies or ladybirds. As far as we know, this study is the first to show that GE or garlicin helps plants recruit natural enemies of aphids.

On the basis of available knowledge, wound-induced, ubiquitous (*Z*)-3-hexenol, a C6-alcohol synthesized in the lipoxygenase/HPL pathway, is the most important infochemical influencing herbivore repellence and attraction in tritrophic interactions^38^. Quiroz and Niemeyer^54^ found that volatiles from wheat and oat seedlings attracted winged and wingless *Rhopalosiphum padi*. These volatiles were identified by GC-MS, and olfactometer tests performed with each compound showed that aphids were attracted by (*E*)-2-hexenyl acetate, (*Z*)-3-hexenol, (*Z*)-2-hexenol and so on. Our result that the Z3H release attracted the highest population densities of *M. dirhodum* and *S. avenae* in (Fig. 2) agrees with their report on the cereal aphid *R. padi*^54^.

The push–pull strategy is a behavioral manipulation method that uses repellent/deterrent (push) and attractive/stimulant (push) stimuli to direct the movement of pest or beneficial insects for pest management^55^. The volatiles tested in the present study were either a repellent or attractant stimuli to aphids and either an attractant or neutral to natural enemies (beneficials), depending on the infochemical. Z3H acted as a pull stimulus to the aphids, but was neutral to beneficials; GE and EBF acted as a push stimulus for the aphids and as a pull for beneficials (EBF to hoverfly, GE to lacewings). The three infochemicals could be used to promote a push–pull strategy and have great potential for integrated pest management of wheat aphids. Recent studies have provided evidence for the potential use of synthetic volatiles as aids to enhance biological control measures in crop ecosystems^13^,^56^,^57^. Targeting the right volatiles for enhanced emission could lead to ecologically and economically sound ways of combating important pests. However, a remaining question surrounding the use of these materials in integrated pest management is the ecological consequences of these synthetic volatiles on predators and parasitoids in the absence of their prey. Therefore, more detailed work on ecological consequences and application rate, dose and duration under field conditions must be done before those volatiles can be developed as a semiochemical tool to replace broad-spectrum insecticides. Manipulating the behavior of natural enemies to improve biological control holds great potential for improving push–pull strategies so that they can be more widely deployed for sustainable agricultural systems in the future.

## Additional Information

**How to cite this article**: Zhou, H. *et al*. Use of slow-release plant infochemicals to control aphids: a first investigation in a Belgian wheat field. *Sci. Rep.* **6**, 31552; doi: 10.1038/srep31552 (2016).

## Figures and Tables

**Table 1:** Total number of aphids and their natural enemies found in yellow traps in different crop systems during 2011 growing season.

Species	Treatments				
	Paraffin oil	(E)-β-Farnesene	Garlic extract	(Z)-3-Hexenol	% of Total^a^
**Aphids**					
*Metopolophum dirhodum* (Walker)	896	585	582	1122	89.5
*Sitobion avenae* (Fabricius)	138	35	54	148	10.5
Diversity and abundance of aphid species %	29.0	17.4	17.9	35.7	
**Ladybirds**	12.8%^b^				
*Harmonia axyridis* Pallas	18	21	22	28	66.8
*Coccinella septempunctata* L.	9	9	8	3	21.8
*Propylea 14-punctata* L.	3	3	1	0	5.3
*Harmonia 4-punctata*	2	1	0	0	2.3
*Calvia 14-guttata*	2	0	0	0	1.5
*Hippodamia variegata* (Goeze)	1	1	0	1	2.3
**Hoverflies**	39.4%^b^				
*Episyrphus balteatus* De Geer	69	108	85	76	82.6
*Scaeva pyrastri* L.	2	0	0	7	2.2
*Sphaerophoria scripta* L.	12	16	9	8	11.0
*Melanostoma scalare* Fabr.	0	3	0	1	1.0
*Metasyrphus corollae* Fabr.	5	1	2	5	3.2
**Lacewings**	47.8%^b^				
*Chrysoperla carnea* Stephens	95	128	152	121	100.0
Total numbers of aphidophagous species	218	291	279	250	
Percentage of total number of aphidophagous species	21.0	28.0	26.9	24.1	

^a^Relative abundance of each species by family.

^b^Relative occurrence of each family in aphidophagous guild.
